# Misclassification of firearm-related violent crime in criminal legal system records: challenges and opportunities

**DOI:** 10.1186/s40621-023-00458-1

**Published:** 2023-10-02

**Authors:** Julia P. Schleimer, Ayah Mustafa, Rachel Ross, Andrew Bowen, Amy Gallagher, Deirdre Bowen, Ali Rowhani-Rahbar

**Affiliations:** 1 Department of Epidemiology, School of Public Health, University of Washington, 3980 15th Ave NE, Seattle, WA 98195 USA; 2 Firearm Injury and Policy Research Program, University of Washington, Seattle, WA USA; 3 School of Law, Seattle University, Seattle, WA USA

**Keywords:** Firearms, Violence, Epidemiologic measurement

## Abstract

**Background:**

Criminal legal system data are one source for measuring some types of firearm-related harms, including those that do not necessarily result in injury or death, but measurement can be hampered by imprecise criminal code statutes. We quantified the degree of misclassification in Washington state criminal codes for measuring firearm-related crime.

**Findings:**

In this study of individuals aged 18 years and older who were convicted of a misdemeanor in Washing­ton Superior Courts from 1/1/2015 through 12/31/2019, we compared firearm-related charges as measured with criminal codes and with manual review of probable cause documents, considered the gold standard. The sample included 5390 criminal cases. Of these, 77 (1.4%) were firearm-related as measured with criminal codes and 437 (8.1%) were firearm-related as measured via manual record review. In the sample overall, the sensitivity of criminal codes was 17.6% (95% CI = 14.2–21.5%), and neg­ative predictive value (NPV) was 93.2% (95% CI = 92.5–93.9%). Sensitivity and NPV were higher for cases with exclusively non-violent charges. For all cases and for cases with any violent crime charge, firearm-related crimes described in probable cause documents most often involved explicit verbal threats, firearm possession, and pointing a firearm at or touching a firearm to someone; almost 13% involved shooting/discharging a firearm. For cases with exclusively non-violent charges, the most com­mon firearm-related crime was unlawful possession.

**Conclusions:**

Criminal records can be used for large-scale policy-relevant studies of firearm-related harms, but this study suggests Washington state criminal codes substantially undercount firearm-related crime, especially firearm-related violent crime.

**Supplementary Information:**

The online version contains supplementary material available at 10.1186/s40621-023-00458-1.

## Main text

Inter-personal firearm violence is a significant and growing public health problem in the United States (US). In 2021, 20,958 Americans died from firearm homicide, reflecting the highest rate since the mid-1990’s and an almost 45% increase from 2019 (Centers for Disease Control and Prevention 2023; Davis et al. 2021). For every person who dies from firearm homicide, many more are non-fatally injured and experience other firearm-related harms, e.g., being threatened or attacked with a firearm, witnessing someone else being shot. Accurately measuring firearm-related harms is critical to preventing them. However, the US currently lacks robust data infrastructure for measuring firearm-related harms other than fatalities (Barber et al. 2022; Roman 2020).

Criminal legal system data are one source for measuring some types of firearm-related harms, including those that do not necessarily result in injury or death. For example, police data capture police-reported incidents in which someone allegedly used a firearm to intentionally injure or threaten another person (Barber et al. 2022). However, police data, which are often de-identified and maintained by individual law enforcement agencies, cannot generally be used to comprehensively link individuals over place and time, and they only reflect one stage in the criminal legal process (Barber et al. 2022; Parker 2022; Roman 2019). Alternatively, records of criminal charges and convictions (hereafter “criminal records”), which are often individually-identified and maintained over time in centralized statewide databases by the state court system, Department of Justice, or other state body, can capture criminalized behavior involving firearms (hereafter “firearm-related crime”) and facilitate longitudinal research on firearm-related criminal charging, bargaining, and sentencing outcomes and the risks of firearm-related crime associated with prior criminal charges, convictions, or other exposures (Kagawa et al. 2020; Wintemute 1998; Rowhani-Rahbar et al. 2015; Swanson et al. 2020). Such research has direct relevance for developing and refining policies and interventions to equitably prevent firearm-related harm, including because convictions (and sometimes charges) form the basis of certain firearm prohibitions (Federal Bureau of Investigation 2019; Bureau of Alcohol, Tobacco, Firearms, and Explosives 2020).

However, the utility of criminal records for measuring firearm-related harm can be hampered by imprecise criminal code statutes that do not distinguish violent crimes that involve firearms from violent crimes that do not involve firearms. That is, because the information available in criminal records is based on state criminal code statutes, and not all statutes differentiate between violent crimes that involve firearms and violent crimes that do not involve firearms, criminal records do not capture firearm-related violent crime in all states (including Washington state, the focus of the current study). Indeed, researchers using criminal records have previously combined violent *or* firearm-related crimes into a single category (in Washington and California) (Rowhani-Rahbar et al. 2015; Wintemute et al. 2018) or used text searching to identify firearm-related violent crimes (in Florida) (Swanson et al. 2020, 2016) due to limitations of criminal codes.

The objective of this retrospective observational study was to quantify the degree of misclassification of firearm-related violent crime with Washington state criminal codes, using manual record review as a gold-standard. Understanding the degree of misclassification bias in criminal codes can inform more rigorous research and development of new coding systems designed to better estimate the true burden of violent firearm-related crime in Washington.

## Methods

### Study setting and population

This was a secondary analysis of data used in a larger cohort study of risk of subsequent firearm-related violent crime associated with downgraded misdemeanor convictions (i.e., initial felony charges downgraded to misdemeanor convictions) (Schleimer et al. 2023). The source population comprised individuals aged 18 years and older who were convicted of a misdemeanor in Washington Superior Courts from 1/1/2015 through 12/31/2019. We identified each individual’s first case during the study that resulted in only misdemeanor convictions (“index conviction”) and then classified each index conviction as downgraded (any initial felony charge) or non-downgraded (initial misdemeanor charges only). We then selected all defendants with non-downgraded convictions and a gender- and age-matched sample of those with downgraded convictions. Matching was done with a propensity score, predicting downgraded convictions based on age and gender, selecting individuals with and without a downgraded conviction in a 4:1 ratio with a nearest-neighbor algorithm. Cohort members were then followed forward in time for new subsequent violent crime charges (misdemeanor or felony) in Washington Superior Courts through 12/31/2020.

The final analytic dataset was at the criminal case level and contained all charges associated with individuals’ index conviction and their first subsequent violent crime charge, if any, during follow-up (i.e., individuals could contribute multiple cases to the analytic data).

Data on criminal charges and convictions were provided by the Washington Administrative Offices of the Courts (AOC) and King County Department of Judicial Administration (KCDJA) and are available to qualified researchers upon request to AOC and KCDJA.

### Violent crime

Violent crimes were measured with the Revised Code of Washington (RCW) criminal codes. The RCW is a collection of Washington state statutes, including criminal statutes, in which the legislature has identified specific behaviors as criminal. In turn, the statutes classify and organize the nature of an alleged criminal action as a specific type of criminal charge, which prosecutors use as a guide to determine what crimes a defendant will be charged with under the RCW. We created two definitions of violent crime using RCW codes (Additional file 1: Table S1). The first definition was restricted to the Federal Bureau of Investigation’s Uniform Crime Reporting (UCR) crimes of murder and nonnegligent manslaughter, forcible rape, robbery, and aggravated assault (hereafter “UCR violent crimes") (Federal Bureau of Investigation 2019)﻿. The second definition included other, non-UCR crimes that reflect an expansive conceptualization of violence (e.g., intimidation, threats, and harassment) and align with the World Health Organization’s definition of violence: “The intentional use of physical force or power, threatened or actual, against oneself, another person, or against a group or community that either results in or has a high likelihood of resulting in injury, death, psychological harm, maldevelopment or deprivation,” (hereafter “non-UCR violent crimes") (Krug et al. 2002).

### Firearm-related crime

Firearm-related charges were measured with both RCW codes and manual record review.

### RCW codes

We classified cases as having a firearm-related charge if criminal records included a firearm-related RCW code (Additional file 1: Table S1). With minor exception, firearm-related RCW codes in Washington only identify non-violent crimes such as theft of a firearm and violations related to firearm possession, carrying, and sales (Additional file 1: Table S1) (Washington State Legislature 2022). They typically do not identify firearm use in violent crimes, e.g., assault. However, if the criminal record indicated the case had a charge for a firearm-related RCW and a violent-related RCW code (described above and in Additional file 1: Table S1), it was classified as a violent firearm-related charge. Cases whose records only included firearm-related RCW codes were classified as non-violent firearm charges.

### Manual record review (“alloyed gold standard”)

To establish an “alloyed gold standard” measure of firearm-related crime (Bodnar et al. 2014), four members of the research team (AM, RR, AB, AG) manually reviewed affidavits of probable cause for each included case obtained from Washington county courts. Affidavits of probable cause include narrative offense descriptions written by law enforcement and are used as evidence to justify an arrest. Because affidavits of probable cause give rise to criminal charges, we considered affidavits to be the most comprehensive measure of firearm-related criminal charges available. Cases were considered firearm-related if the narrative description indicated that the defendant allegedly possessed or used a firearm during the commission of a crime, made firearm-related threats, or was in violation of any firearm-related laws. Cases clearly involving a non-firearm gun (e.g., BB gun) were coded as non-firearm-related per Washington state law, RCW 9.41.010(12). Team members were instructed to be inclusive in their initial decisions. Another team member (JPS) reviewed each affirmative decision. Discrepancies and questions were resolved during team meetings. A case was classified as violent firearm-related if manual review indicated it was firearm-related and the criminal record included a violent-related RCW code (described above and in Additional file 1: Table S1).

### Thematic coding of firearm-related crime

After identifying firearm-related cases via manual review, three members of the research team (JPS, AM, RR) used an inductive process to thematically code each case based on the probable cause narratives into non-mutually exclusive categories for descriptive purposes. Categories were refined during the coding process, and questions were discussed during team meetings. See Additional file 1: Table S2 for a description and example of each category.

### Analysis

We compared classifications of firearm-related crime based on criminal codes and manual record review after restricting our sample to cases in which we had complete data on firearm-related crime from both sources (excluding 155 cases in which affidavits of probable cause were unavailable). While we had information on criminal charges and convictions, we focused on RCW codes recorded in criminal charges rather than convictions since convictions may not correspond well to affidavits of probable cause for reasons other than misclassification, e.g., because of judge or jury decision making and other criminal legal system processes such as plea bargaining.

We calculated overall and violent crime-stratified measures of sensitivity and negative predictive value (NPV) by constructing a 2 × 2 validation table, cross-classifying cases according to the two measurement sources (Fox et al. 2020). Considering manual record review as the “truth,” sensitivity is the proportion of truly firearm-related cases, i.e., firearm positive, that are classified as firearm-related by criminal codes (i.e., true positives divided by the sum of true positives and false negatives). Higher sensitivity is desirable and indicates that a larger share of true positives was correctly classified by the non-gold standard measure. NPV is the proportion of cases that are classified as non-firearm-related, i.e., firearm negative, by criminal codes that are truly not firearm-related (i.e., true negatives divided by the sum of true negatives and false negatives). Higher NPV is likewise desirable and indicates that the non-gold standard measure correctly classified a larger share of true negatives.

Specificity, i.e., the proportion of truly non-firearm-related cases that are classified as non-firearm-related by criminal codes, and positive predictive value, i.e., the proportion of cases that are classified as firearm-related by criminal codes that are truly firearm-related, were 100% in this context and thus not reported.

To gain additional insight into the types of firearm-related crimes that were misclassified and inform bias analyses of specific crime types, we presented stratified measures for cases with any UCR violent crime charge, specific UCR violent crime charges, any non-UCR violent crime charge, and exclusively non-violent charges (neither UCR-violent crime charges nor non-UCR violent crime charges). We also described the frequency of firearm-related crime types per our thematic coding.

We used Research Electronic Data Capture (RedCap), hosted at the Institute of Translational Health Sciences (ITHS) (Harris et al. 2009), and Microsoft excel for data collection and management and R version 4.0.0 (R Foundation for Statistical Computing, Vienna, Austria) for data analysis. Exact 95% confidence intervals were constructed for proportions using the epi.conf function in the epiR package (version 2.0.38) (Stevenson et al. 2023). The University of Washington Institutional Review Board approved this study.

## Results

The sample included 5,390 criminal cases. Of these, 77 (1.4%) were firearm-related as measured with criminal codes and 437 (8.1%) were firearm-related as measured via manual record review (Table 1). In the sample overall, the sensitivity of criminal codes was 17.6% (95% CI 14.2–21.5%), and NPV was 93.2% (95% CI 92.5–93.9%). Sensitivity (17.6%; 95% CI 10.9–26.1%) and NPV (90.8%; 95% CI 88.8–92.6%) were similar for cases with one or more UCR violent crime charges (Table 2). For cases with one or more non-UCR violent crime charges, sensitivity was lower, at 10.4% (95% CI 7.1–14.5%), and NPV was similar, at 90.2% (95% CI 89.0–91.3%)(Table 3). For cases with exclusively non-violent charges, sensitivity was 45.9% (95% CI 34.3–57.9%) and NPV was 98.0% (95% CI 97.3–98.6%) (Table 4).

**Table 1:** Sensitivity and negative predictive value of firearm-related cases, all cases

		Manual review			
		No. Firearm-Related	No. Not Firearm-Related	Total	
Criminal Codes	No. Firearm-Related	77	0	77	
	No. Not Firearm-Related	360	4953	5313	NPV = 93.2% (95% CI 92.5–93.9%)
	Total	437	4953	5390	
		Se = 17.6% (95% CI 14.2–21.5%)			

Se, Sensitivity; NPV, Negative Predictive Value; No., number

**Table 2:** Sensitivity and negative predictive value of firearm-related cases with one or more UCR violent crime charge

		Manual review			
		No. Firearm-Related	No. Not Firearm-Related	Total	
Criminal Codes	No. Firearm-Related	19	0	19	
	No. Not Firearm-Related	89	882	971	NPV = 90.8% (95% CI 88.8–92.6%)
	Total	108	882	990	
		Se = 17.6% (95% CI 10.9–26.1%)			

Se, Sensitivity; NPV, Negative Predictive Value; No., number

**Table 3:** Sensitivity and negative predictive value of firearm-related cases with one or more non-UCR violent crime charge

		Manual Review			
		No. Firearm-Related	No. Not Firearm-Related	Total	
Criminal Codes	No. Firearm-Related	30	0	30	
	No. Not Firearm-Related	258	2366	2624	NPV = 90.2% (95% CI 89.0–91.3%)
	Total	288	2366	2654	
		Se = 10.4% (95% CI 7.1–14.5%)			

Se, Sensitivity; NPV, Negative Predictive Value; No., number

**Table 4:** Sensitivity and negative predictive value of firearm-related cases with exclusively non-violent charges

		Manual Review			
		No. Firearm-Related	No. Not Firearm-Related	Total	
Criminal Codes	No. Firearm-Related	34	0	34	
	No. Not Firearm-Related	40	1946	1986	NPV = 98.0% (95% CI 97.3–98.6%)
	Total	74	1946	2020	
		Se = 45.9% (95% CI 34.3–57.9%)			

Se, Sensitivity; NPV, Negative Predictive Value; No., number

Additional file 1: Tables S3–S5 show sensitivity and NPV for three specific categories of UCR violent crimes: (1) murder, non-negligent homicide, aggravated assault (these were combined due to the small number of murders/homicides in our sample) (Additional file 1: Table S3), (2) robbery (Additional file 1: Table S4), and (3) rape (Additional file 1: Table S5). Sensitivity was highest for murder, non-negligent homicide, and aggravated assault (19.4%; 95% CI 11.9–28.9%) and NPV was highest for rape (97.2%, 95% CI 85.5–99.9%).

For all cases combined, cases with any UCR violent crime charge, and cases with any non-UCR violent crime charge, types of firearm-related crimes most often described in affidavits of probable cause were explicit verbal threats, firearm possession, and pointing a firearm at or touching a firearm to someone (Table 5). Shooting or discharging a firearm was also common among fire­arm-related cases with any UCR violent crime charges (23.1%). For firearm-related cases with exclusively non-violent charges, the most common types of firearm-related crimes were unlawful possession (43.2%) and firearm possession (36.5%).

**Table 5:** Description of types of firearm-related behavior identified in manual record review

Category^a^	No. (%)
**All Cases**	N = 437 (100)
Explicit verbal threat	200 (45.8)
Explicit written threat	16 (3.7)
Explicit verbal or written threat	46 (10.5)
Threatened-unknown verbal/written/action	7 (1.6)
Shooting/discharge^b^	56(12.8)
Intentionally shot and hit person	8 (1.8)
Intentionally shot person but did not hit person	18 (4.1)
Unintentionally shot and hit person	4 (0.9)
Intentionally shot non-person but hit person	2 (0.5)
Intentionally shot at non-person, did not hit person	14 (3.2)
Intentionally shot around person, did not hit person	7 (1.6)
Intentionally shot animal	3 (0.7)
Physical harm	8 (1.8)
Point/touch firearm	102 (23.3)
Brandish	57 (13.0)
Other firearm threat	38 (8.7)
Hunting	9 (2.1)
Unlawful possession	55 (12.6)
Firearm possession	145 (33.2)
Stole firearm	15 (3.4)
**Cases With > = 1 UCR Violent Crime Charges**	N = 108 (100)
Explicit verbal threat	42 (38.9)
Explicit written threat	2 (1.9)
Explicit verbal or written threat	5 (4.6)
Threatened-unknown verbal/written/action	1 (0.9)
Shooting/discharge^b^	25 (23.1)
Intentionally shot and hit person	7 (6.5)
Intentionally shot person but did not hit person	8 (7.4)
Unintentionally shot and hit person	3 (2.8)
Intentionally shot non-person but hit person	0 (0)
Intentionally shot at non-person, did not hit person	4 (3.7)
Intentionally shot around person, did not hit person	3 (2.8)
Intentionally shot animal	0 (0)
Physical harm	6 (5.6)
Point/touch firearm	60 (55.6)
Brandish	18 (16.7)
Other firearm threat	6 (5.6)
Hunting	1 (0.9)
Unlawful possession	8 (7.4)
Firearm possession	56 (51.9)
Stole firearm	1 (0.9)
**Cases With > = 1 Non-UCR Violent Crime Charges**	N = 288 (100)
Explicit verbal threat	167 (58.0)
Explicit written threat	15 (5.2)
Explicit verbal or written threat	45 (15.6)
Threatened-unknown verbal/written/action	6 (2.1)
Shooting/discharge^b^	25 (8.7)
Intentionally shot and hit person	1 (0.3)
Intentionally shot person but did not hit person	9 (3.1)
Unintentionally shot and hit person	0 (0)
Intentionally shot non-person but hit person	2 (0.7)
Intentionally shot at non-person, did not hit person	9 (3.1)
Intentionally shot around person, did not hit person	4 (1.4)
Intentionally shot animal	0 (0)
Physical harm	5 (1.7)
Point/touch firearm	55 (19.1)
Brandish	36 (12.5)
Other firearm threat	31 (10.8)
Hunting	0 (0)
Unlawful possession	18 (6.3)
Firearm possession	78 (27.1)
Stole firearm	3 (1.0)
**Cases With Exclusively Non-Violent Charges**	N = 74 (100)
Explicit verbal threat	6 (8.1)
Explicit written threat	0 (0)
Explicit verbal or written threat	0 (0)
Threatened-unknown verbal/written/action	0 (0)
Shooting/discharge^b^	13 (17.6)
Intentionally shot and hit person	1 (1.4)
Intentionally shot person but did not hit person	4 (5.4)
Unintentionally shot and hit person	1 (1.4)
Intentionally shot non-person but hit person	0 (0)
Intentionally shot at non-person, did not hit person	3 (4.1)
Intentionally shot around person, did not hit person	1 (1.4)
Intentionally shot animal	3 (4.1)
Physical harm	1 (1.4)
Point/touch firearm	3 (4.1)
Brandish	7 (9.5)
Other firearm threat	2 (2.7)
Hunting	8 (10.8)
Unlawful possession	32 (43.2)
Firearm possession	27 (36.5)
Stole firearm	11 (14.9)

^a^Categories are not mutually exclusive per case

^b^Sub-categories of shooting/discharge are mutually exclusive

## Discussion

This study quantified the degree to which Washington state criminal codes undercount firearm-related crime as reflected in criminal charges. The sensitivity of Washington state criminal codes was consistently below 20% when examining all cases together and when focusing on cases that only involved violence. As expected, sensitivity was higher (almost 50%) for non-violent crime. Depending on the source used, the percentage of alleged crimes in our sample that were firearm-related ranged from 1.4 to 8.1%, and the percentage of alleged violent crimes that were firearm-related ranged from 0 to 13.6%, depending on the specific definition/sub-categories of violence used. Approximately 7–8% of all violent victimizations reported in the National Crime Victimization Survey in 2018–2019 involved a firearm, similar to our estimates based on manual review and much higher than our estimates based on criminal codes (Morgan and Truman 2019). Of offenses in the western US reported to the FBI in 2019, a larger percent of murders (65.9%), robberies (26.4%) and aggravated assaults (19.9%) involved firearms than our manual review estimates reflect (Federal Bureau of Investigation 2019); this is likely because our sample primarily comprised misdemeanor cases, which are lower-level cases than felonies.

By quantifying this misclassification at the charging stage, our results can improve the rigor and validity of studies that use criminal records to study firearm-related harm. First, our sensitivity estimates can inform quantitative bias analyses for misclassification of firearm-related crimes in other studies in which criminal codes do not distinguish between firearm-related and non-firearm-related violent crimes and in which manual record review is unfeasible (Lash et al. 2014). Negative predictive value depends on setting-specific prevalence so may not be similarly transportable, meaning our estimates of NPV may not generalize to other settings in which the true prevalence of firearm-related crime differs from our study (Fox et al. 2020).

In settings in which our estimates do not generalize or deterministic individual-level assessment of firearm-related crime is needed, our study demonstrates that court records contain valuable information for measuring criminalized behavior involving firearms. Natural language processing tools can be developed to classify cases as firearm-related or non-firearm-related based on the narrative text in affidavits of probable cause or potentially other court documents; similar approaches have been used in other areas of violence prevention research (Kafka et al. 2023). Such automated processes would reduce the human resources needed for manual record review and allow use of court records for firearm-related crime identification to be implemented at scale.

Third, our results suggest that criminal statutes in Washington state and other states with similar coding systems should be revised to better capture alleged firearm-related crime. Such revision has precedent. For example, in the 1980’s, California Penal Code Section 245 was amended to identify firearm use in assaults (Justia Law 1989﻿). As efforts to link and harmonize criminal legal system data across states continue, revising state criminal statutes to better identify firearm-related violent crime in criminal records would have substantial value (Institute for Social Research University of Michigan 2023). Importantly, revising criminal code statutes need not further criminalize behaviors that are not currently criminalized; rather, criminal code statutes need only be revised to more clearly classify existing crimes as to whether or not they involved a firearm. For example, rather than the current catch-all “deadly weapon” language, RCW codes could be modified to differentiate firearm vs. non-firearm weapons.

Our study has several limitations. Manual record review of affidavits of probable cause may itself have measurement error arising from inaccurate or incomplete documentation by police or from errors in coding. For example, we were not always able to confirm whether probable cause affidavits referred to firearms or non-firearm guns since terms such as “gun” and “shoot” are often used to refer to firearms (and we coded them as such) but may also be used in reference to non-firearm guns. However, we did not code cases as firearm-related if the evidence clearly suggested the crime involved a non-firearm gun (e.g., BB gun), consistent with Washington state law, RCW 9.41.010(12). We were missing 155 records. Affidavits of probable cause might differ from formal criminal charges for reasons other than measurement error (i.e., police documentation, prosecutor discretion in charging), so our estimates of sensitivity likely reflect a lower bound. Additionally, as mentioned above, sensitivity may be affected if our sample disproportionately over- or under-included those with criminal code-measured firearm-related charges. However, the proportion of firearm-related charges measured with criminal codes did not differ between the overall population (1.5%) and the population sampled based on downgraded convictions (1.4%). Therefore, the marginal distribution of firearm-related crimes measured via manual record review should not differ from the target population, and our estimates of sensitivity will be approximately valid (Fox et al. 2020). Finally, we recognize that there can be substantial differences between actual criminalized behavior, documented probable cause that a crime was committed, charges brought by a prosecutor, and an ultimate conviction. This analysis compared two of several possible measures of criminal legal system-documented criminalized behavior with firearms. Our results may not generalize to other stages in the criminal legal system (e.g., convictions) or other settings with different criminal coding systems. These are areas for future research.

## Conclusions

Criminal records can be used to conduct large-scale policy-relevant studies of firearm-related harms, but our results suggest that Washington state criminal codes substantially undercount firearm-related crime as reflected in criminal charges, especially firearm-related violent crime. This study can be used to inform quantitative bias analyses of firearm-related crime, novel approaches to firearm-related crime identification, and motivate modifications of state criminal coding systems.

## Supplementary Information

**Additional file 1**. Suplementary methodological information and results.

## Data Availability

The data that support the findings of this study are available from the Washington Administrative Offices of the Courts and King County Department of Judicial Administration but restrictions apply to the availability of these data, which were used under license for the current study, and so are not publicly available.
