# Correction: Misclassification of firearm-related violent crime in criminal legal system records: challenges and opportunities

**DOI:** 10.1186/s40621-025-00605-w

**Published:** 2025-09-18

**Authors:** Julia P. Schleimer, Ayah Mustafa, Rachel Ross, Andrew Bowen, Amy Gallagher, Deirdre Bowen, Ali Rowhani‑Rahbar

**Affiliations:** 1 Department of Epidemiology, School of Public Health, University of Washington, 3980 15th Ave NE, Seattle, WA 98195 USA; 2 Firearm Injury and Policy Research Program, University of Washington, Seattle, WA USA; 3 School of Law, Seattle University, Seattle, WA USA

**Correction:** **Injury Epidemiology** **(2023) 10:46 **

10.1186/s40621-023-00458-1

Following publication of the original article [1], the authors identified a minor coding error that slightly changes the distribution of the sample presented in Table 5 (Description of types of firearm-related behavior identified in manual record review). The correction minimally affects the summary distribution (numbers and percentages) only in that table and has no effect on the interpretation and conclusion of the article. 

The findings section in the abstract reads:

**Findings**: In this study of individuals aged 18 years and older who were convicted of a misdemeanor in Washington Superior Courts from 1/1/2015 through 12/31/2019, we compared firearm-related charges as measured with criminal codes and with manual review of probable cause documents, considered the gold standard. The sample included 5,390 criminal cases. Of these, 77 (1.4%) were firearm-related as measured with criminal codes and 437 (8.1%) were firearm-related as measured via manual record review. In the sample overall, the sensitivity of criminal codes was 17.6% (95% CI = 14.2–21.5%), and negative predictive value (NPV) was 93.2% (95% CI = 92.5–93.9%). Sensitivity and NPV were higher for cases with exclusively non-violent charges. For all cases and for cases with any violent crime charge, firearm-related crimes described in probable cause documents most often involved explicit verbal threats, firearm possession, and pointing a firearm at or touching a firearm to someone; almost 10% involved shooting/discharging a firearm. For cases with exclusively non-violent charges, the most common firearm-related crime was unlawful possession.

The findings section in the abstract should instead read:

**Findings**: In this study of individuals aged 18 years and older who were convicted of a misdemeanor in Washington Superior Courts from 1/1/2015 through 12/31/2019, we compared firearm-related charges as measured with criminal codes and with manual review of probable cause documents, considered the gold standard. The sample included 5,390 criminal cases. Of these, 77 (1.4%) were firearm-related as measured with criminal codes and 437 (8.1%) were firearm-related as measured via manual record review. In the sample overall, the sensitivity of criminal codes was 17.6% (95% CI = 14.2–21.5%), and negative predictive value (NPV) was 93.2% (95% CI = 92.5–93.9%). Sensitivity and NPV were higher for cases with exclusively non-violent charges. For all cases and for cases with any violent crime charge, firearm-related crimes described in probable cause documents most often involved explicit verbal threats, firearm possession, and pointing a firearm at or touching a firearm to someone; almost 13% involved shooting/discharging a firearm. For cases with exclusively non-violent charges, the most common firearm-related crime was unlawful possession.

The last paragraph of the results section reads:

For all cases combined, cases with any UCR violent crime charge, and cases with any non-UCR violent crime charge, types of firearm-related crimes most often described in affidavits of probable cause were explicit verbal threats, firearm possession, and pointing a firearm at or touching a firearm to someone (Table 5). Shooting or discharging a firearm was also common among firearm-related cases with any UCR violent crime charges (19.4%). For firearm-related cases with exclusively non-violent charges, the most common types of firearm-related crimes were unlawful possession (30.7%) and firearm possession (21.6%)

The last paragraph of the results section should read:

For all cases combined, cases with any UCR violent crime charge, and cases with any non-UCR violent crime charge, types of firearm-related crimes most often described in affidavits of probable cause were explicit verbal threats, firearm possession, and pointing a firearm at or touching a firearm to someone (Table 5). Shooting or discharging a firearm was also common among firearm-related cases with any UCR violent crime charges (23.1%). For firearm-related cases with exclusively non-violent charges, the most common types of firearm-related crimes were unlawful possession (43.2%) and firearm possession (36.5%). 

Table 5 currently reads:

**Table:** Table 5. Description of types of firearm-related behavior identified in manual record review

Category^a^	No. (%)
*All Cases*	*N* = 437 (100)
Explicit verbal threat	139 (31.8)
Explicit written threat	9 (2.1)
Explicit verbal or written threat	33 (7.6)
Threatened-unknown verbal/written/action	6 (1.4)
Shooting/discharge^b^	41 (9.4)
o Intentionally shot and hit person	7 (1.6)
o Intentionally shot person but did not hit person	12 (2.7)
o Unintentionally shot and hit person	2 (0.5)
o Intentionally shot non-person but hit person	2 (0.5)
o Intentionally shot at non-person, did not hit person	10 (2.3)
o Intentionally shot around person, did not hit person	6 (1.4)
o Intentionally shot animal	2 (0.5)
Physical harm	6 (1.4)
Point/touch firearm	73 (16.7)
Brandish	37 (8.5)
Other firearm threat	27 (6.2)
Hunting	8 (1.8)
Unlawful possession	40 (9.2)
Firearm possession	98 (22.4)
Stole firearm	7 (1.6)
*Cases With > = 1 UCR Violent Crime Charges*	*N* = 108 (100)
Explicit verbal threat	30 (27.8)
Explicit written threat	1 (0.9)
Explicit verbal or written threat	4 (3.7)
Threatened-unknown verbal/written/action	1 (0.9)
Shooting/discharge^b^	21 (19.4)
o Intentionally shot and hit person	6 (5.6)
o Intentionally shot person but did not hit person	7 (6.5)
o Unintentionally shot and hit person	2 (1.9)
o Intentionally shot non-person but hit person	0 (0)
o Intentionally shot at non-person, did not hit person	3 (2.8)
o Intentionally shot around person, did not hit person	3 (2.8)
o Intentionally shot animal	0 (0)
Physical harm	4 (3.7)
Point/touch firearm	44 (40.7)
Brandish	13 (12)
Other firearm threat	5 (4.6)
Hunting	1 (0.9)
Unlawful possession	6 (5.6)
Firearm possession	44 (40.7)
Stole firearm	1 (0.9)
*Cases With > = 1 Non-UCR Violent Crime Charges*	*N* = 288 (100)
Explicit verbal threat	113 (39.2)
Explicit written threat	9 (3.1)
Explicit verbal or written threat	32 (11.2)
Threatened-unknown verbal/written/action	5 (1.7)
Shooting/discharge^b^	19 (6.6)
o Intentionally shot and hit person	1 (0.3)
o Intentionally shot person but did not hit person	6 (2.1)
o Unintentionally shot and hit person	0 (0)
o Intentionally shot non-person but hit person	2 (0.7)
o Intentionally shot at non-person, did not hit person	7 (2.4)
o Intentionally shot around person, did not hit person	3 (1.0)
o Intentionally shot animal	0 (0)
Physical harm	4 (1.4)
Point/touch firearm	39 (13.5)
Brandish	23 (8)
Other firearm threat	21 (7.3)
Hunting	0 (0)
Unlawful possession	13 (4.5)
Firearm possession	51 (17.7)
Stole firearm	1 (0.3)
*Cases With Exclusively Non-Violent Charges*	*N* = 74 (100)
Explicit verbal threat	5 (6.7)
Explicit written threat	0 (0)
Explicit verbal or written threat	0 (0)
Threatened-unknown verbal/written/action	0 (0)
Shooting/discharge^b^	8 (10.8)
o Intentionally shot and hit person	1 (1.3)
o Intentionally shot person but did not hit person	2 (2.7)
o Unintentionally shot and hit person	0 (0)
o Intentionally shot non-person but hit person	0 (0)
o Intentionally shot at non-person, did not hit person	2 (2.7)
o Intentionally shot around person, did not hit person	1 (1.3)
o Intentionally shot animal	2 (2.7)
Physical harm	1 (1.3)
Point/touch firearm	1 (1.3)
Brandish	3 (4)
Other firearm threat	2 (2.7)
Hunting	7 (9.3)
Unlawful possession	23 (30.7)
Firearm possession	16 (21.6)
Stole firearm	5 (6.7)

^a^Categories are not mutually exclusive per case

^b^Sub-categories of shooting/discharge are mutually exclusive

Table 5 should instead read:

**Table 5:** Description of types of firearm-related behavior identified in manual record review

Category^a^	No. (%)
**All Cases**	N=437 (100)
Explicit verbal threat	200 (45.8)
Explicit written threat	16 (3.7)
Explicit verbal or written threat	46 (10.5)
Threatened-unknown verbal/written/action	7 (1.6)
Shooting/discharge^b^	56 (12.8)
o Intentionally shot and hit person	8 (1.8)
o Intentionally shot person but did not hit person	18 (4.1)
o Unintentionally shot and hit person	4 (0.9)
o Intentionally shot non-person but hit person	2 (0.5)
o Intentionally shot at non-person, did not hit person	14 (3.2)
o Intentionally shot around person, did not hit person	7 (1.6)
o Intentionally shot animal	3 (0.7)
Physical harm	8 (1.8)
Point/touch firearm	102 (23.3)
Brandish	57 (13.0)
Other firearm threat	38 (8.7)
Hunting	9 (2.1)
Unlawful possession	55 (12.6)
Firearm possession	145 (33.2)
Stole firearm	15 (3.4)
**Cases With >=1 UCR Violent Crime Charges**	N=108 (100)
Explicit verbal threat	42 (38.9)
Explicit written threat	2 (1.9)
Explicit verbal or written threat	5 (4.6)
Threatened-unknown verbal/written/action	1 (0.9)
Shooting/discharge^b^	25 (23.1)
o Intentionally shot and hit person	7 (6.5)
o Intentionally shot person but did not hit person	8 (7.4)
o Unintentionally shot and hit person	3 (2.8)
o Intentionally shot non-person but hit person	0 (0)
o Intentionally shot at non-person, did not hit person	4 (3.7)
o Intentionally shot around person, did not hit person	3 (2.8)
o Intentionally shot animal	0 (0)
Physical harm	6 (5.6)
Point/touch firearm	60 (55.6)
Brandish	18 (16.7)
Other firearm threat	6 (5.6)
Hunting	1 (0.9)
Unlawful possession	8 (7.4)
Firearm possession	56 (51.9)
Stole firearm	1 (0.9)
**Cases With >=1 Non-UCR Violent Crime Charges**	N=288 (100)
Explicit verbal threat	167 (58.0)
Explicit written threat	15 (5.2)
Explicit verbal or written threat	45 (15.6)
Threatened-unknown verbal/written/action	6 (2.1)
Shooting/discharge^b^	25 (8.7)
o Intentionally shot and hit person	1 (0.3)
o Intentionally shot person but did not hit person	9 (3.1)
o Unintentionally shot and hit person	0 (0)
o Intentionally shot non-person but hit person	2 (0.7)
o Intentionally shot at non-person, did not hit person	9 (3.1)
o Intentionally shot around person, did not hit person	4 (1.4)
o Intentionally shot animal	0 (0)
Physical harm	5 (1.7)
Point/touch firearm	55 (19.1)
Brandish	36 (12.5)
Other firearm threat	31 (10.8)
Hunting	0 (0)
Unlawful possession	18 (6.3)
Firearm possession	78 (27.1)
Stole firearm	3 (1.0)
**Cases With Exclusively Non-Violent Charges**	N=74 (100)
Explicit verbal threat	6 (8.1)
Explicit written threat	0 (0)
Explicit verbal or written threat	0 (0)
Threatened-unknown verbal/written/action	0 (0)
Shooting/discharge^b^	13 (17.6)
o Intentionally shot and hit person	1 (1.4)
o Intentionally shot person but did not hit person	4 (5.4)
o Unintentionally shot and hit person	1 (1.4)
o Intentionally shot non-person but hit person	0 (0)
o Intentionally shot at non-person, did not hit person	3 (4.1)
o Intentionally shot around person, did not hit person	1 (1.4)
o Intentionally shot animal	3 (4.1)
Physical harm	1 (1.4)
Point/touch firearm	3 (4.1)
Brandish	7 (9.5)
Other firearm threat	2 (2.7)
Hunting	8 (10.8)
Unlawful possession	32 (43.2)
Firearm possession	27 (36.5)
Stole firearm	11 (14.9)

^a^Categories are not mutually exclusive per case

^b^Sub-categories of shooting/discharge are mutually exclusive

The Findings in the abstract, the last paragraph of the results section and Table 5 have been updated in this correction and the original article [1] has been corrected.
