# Supplementary material for: Misclassification of firearm-related violent crime in criminal legal system records: challenges and opportunities
Source: Inj Epidemiol. 2023 Oct 2;10:46. doi: 10.1186/s40621-023-00458-1 (PMC10544360; doi:10.1186/s40621-023-00458-1)
Supplement: Supplementary file 1 — Additional file 1. Suplementary methodological information and results. [file 40621_2023_458_MOESM1_ESM.docx]

**Supplement**

Misclassification of Firearm-Related Violent Crime in Criminal Legal System Records: Challenges and Opportunities

Julia P. Schleimer, MPH,^1,2^ Ayah Mustafa, BS,^2^ Rachel Ross, MPH,^2^ Andrew Bowen, BA,^2^ Amy Gallagher, MPH,^2^ Deirdre Bowen, JD, PhD,^2,3^ Ali Rowhani-Rahbar, MD, PhD, MPH^1,2^

^1^Department of Epidemiology, School of Public Health, University of Washington, Seattle, WA, USA

^2^Firearm Injury and Policy Research Program, University of Washington, Seattle, WA, USA

^3^School of Law, Seattle University, Seattle, WA, USA

Supplementary Table 1. Revised Code of Washington (RCW) Codes for Violent and Firearm-Related Charges Among Study Cohort (N=5390 Cases)

| **RCW** | **Description** | **UCR Violent^a^** | **Non-UCR Violent^b^** | **Firearm-Related** |
| --- | --- | --- | --- | --- |
| 9A.32.030(1)(A) | MURDER-1 PREMEDITATED | x |  |  |
| 9A.32.030(1)(B) | MURDER-1 EXTREME INDIF HUMAN LIFE | x |  |  |
| 9A.32.030(1)(C) | MURDER-1 IN COURSE OF OTHER CRIME | x |  |  |
| 9A.32.050 | MURDER-2 | x |  |  |
| 9A.32.050(1)(A) | MURDER-2 NO PREMED/DEATH OF ANTHR | x |  |  |
| 9A.32.050(1)(B) | MURDER-2 FELONY/ASSAULT | x |  |  |
| 9A.36.011 | ASSAULT-1 | x |  |  |
| 9A.36.011(1)(A) | ASSAULT-1 DEADLY WEAPON/FORCE | x |  |  |
| 9A.36.011(1)(C) | ASSAULT-1 BODILY HARM | x |  |  |
| 9A.36.021(1) | ASSAULT-2 | x |  |  |
| 9A.36.021(1)(A) | ASSAULT-2 SUBSTANTIAL BODILY HARM | x |  |  |
| 9A.36.021(1)(C) | ASSAULT-2 DEADLY WEAPON | x |  |  |
| 9A.36.021(1)(D) | ASSAULT 2ND DEG (POISON/SUBSTANCE) | x |  |  |
| 9A.36.021(1)(E) | ASSAULT-2 INTENT TO COMMIT FELONY | x |  |  |
| 9A.36.021(1)(G) | ASSAULT-2 STRANGULATION | x |  |  |
| 9A.36.021(2)(A) | ASSAULT-2 PENALTY | x |  |  |
| 9A.36.021(2)(B) | ASSAULT-2 SEX MOTIVATION PENALTY | x |  |  |
| 9A.36.130 | ASSAULT OF CHILD-2 | x |  |  |
| 9A.44.040(1)(D) | RAPE-1 FELONIOUS ENTER BUILDING/VEH | x |  |  |
| 9A.44.050 | RAPE-2 | x |  |  |
| 9A.44.050(1)(A) | RAPE-2 BY FORCIBLE COMPULSION | x |  |  |
| 9A.44.050(1)(B) | RAPE-2 INCAPABLE OF CONSENT | x |  |  |
| 9A.44.073 | RAPE OF A CHILD-1 | x |  |  |
| 9A.44.076 | RAPE OF A CHILD-2 | x |  |  |
| 9A.44.076(2) | RAPE OF A CHILD-2 PENALTY | x |  |  |
| 9A.56.200 | ROBBERY-1 | x |  |  |
| 9A.56.200(1)(A) | ROBBERY-1 | x |  |  |
| 9A.56.200(1)(B) | ROBBERY-1 W/IN FINANCIAL INSTITUTION | x |  |  |
| 9A.56.200(1)A1 | ROBBERY-1 DEADLY WEAPON | x |  |  |
| 9A.56.200(1)A2 | ROBBERY-1 DISPLAY FIREARM/ DEADLY WEAPON | x |  |  |
| 9A.56.200(1)A3 | ROBBERY-1 INFLICT BODY INJURY | x |  |  |
| 9A.56.210 | ROBBERY-2 | x |  |  |
| 46.61.520 | VEHICULAR HOMICIDE |  | x |  |
| 46.61.520(1)(A) | VEH HOMICIDE-INFLUENCE ALCOHOL/DRUG |  | x |  |
| 46.61.520(1)(B) | VEHICULAR HOMICIDE-RECKLESS |  | x |  |
| 46.61.520(1)(C) | VEHICULAR HOMICIDE-DISREGARD SAFETY |  | x |  |
| 46.61.522 | VEHICULAR ASSAULT |  | x |  |
| 46.61.522(1)(A) | VEHICULAR ASSAULT-RECKLESS |  | x |  |
| 46.61.522(1)(B) | VEHICULAR ASSAULT-DRIVE UND INFL |  | x |  |
| 46.61.522(1)(C) | VEHICULAR ASSAULT-DISREGARD SAFETY |  | x |  |
| 9A.32.060(1)(A) | MANSLAUGHTER-1 RECKLESS |  | x |  |
| 9A.32.070 | MANSLAUGHTER-2 |  | x |  |
| 10.14.120 | ANTI-HARASSMENT/PROT TEMP ORDER VIOLATION |  | x |  |
| 10.14.170 | VIOLATE ANTI-HARASSMENT--PENALTY |  | x |  |
| 26.50.110(1) | PROTECTION ORDER VIOLATION (GM) |  | x |  |
| 26.50.110(4) | PROTECTION ORDER VIOLATION-FELONY |  | x |  |
| 26.50.110(5) | PROTECTION ORDER VIOLATION-PREV CONV |  | x |  |
| 46.52.020(1) | DUTY OF DRIVER-VEHICLE ACCIDENT |  | x |  |
| 46.52.020(4)(B) | HIT/RUN ACCIDENT-INJURY |  | x |  |
| 9.41.230 | AIMING OR DISCHARGING FIREARMS/WEAPON |  | x |  |
| 9.41.230(1)(A) | AIM FIREARM AT PERSON |  | x | x |
| 9.41.230(1)(B) | DISCHARGE FIREARM/WEAPON PUBLIC PLACE |  | x |  |
| 9.41.230(1)(C) | AIM/DISCHARGE WEAPON |  | x |  |
| 9.41.270 | WEAPONS CAPABLE PRODUCING HARM |  | x |  |
| 9.41.270(1) | WEAPON CAPABLE PRODUCE HARM DISPLAY |  | x |  |
| 9.61.160(1) | BOMB THREAT-INTENT TO ALARM |  | x |  |
| 9.61.230(1) | TELEPHONE HARASSMENT |  | x |  |
| 9.61.230(2)(B) | TELEPHONE-THREATS TO KILL |  | x |  |
| 9.61.260(1) | CYBERSTALKING |  | x |  |
| 9.61.260(2) | CYBERSTALKING |  | x |  |
| 9.61.260(3) | CYBERSTALKING PREV CONV/DEATH THREAT |  | x |  |
| 9.61.260(3)(B) | CYBERSTALKING THREATEN TO KILL |  | x |  |
| 9.62.010(1) | MALICIOUS PROSECUTION OF FEL PEN |  | x |  |
| 9.68A.040 | SEXUAL EXPLOITATION OF A MINOR |  | x |  |
| 9.68A.050(1) | DEAL DEPICT MINOR-SEX CONDUCT-1 |  | x |  |
| 9.68A.050(2) | DEAL DEPICT MINOR-SEX CONDUCT-2 |  | x |  |
| 9.68A.070(1)(A) | POSSESS DEPICT MINOR-SEX CONDUCT-1 |  | x |  |
| 9.68A.070(2) | POSSESS DEPICT MINOR-SEX CONDUCT-2 |  | x |  |
| 9.68A.070(2)(A) | POSSESS DEPICT MINOR-SEX CONDUCT-2 |  | x |  |
| 9.68A.090(1) | COMMUNICATION W/MINOR-IMMORAL PURPOSES |  | x |  |
| 9.68A.090(2) | COMMUNICATION W/MINOR-IMMORAL PREVIOUS CONVICTION |  | x |  |
| 9.68A.100 | COMMERCIAL SEX ABUSE OF A MINOR |  | x |  |
| 9.68A.100(1)(B) | COMMERCIAL SEX ABUSE OF A MINOR |  | x |  |
| 9.94.010(1) | PRISON RIOT-PARTICIPATE/AID/ABET |  | x |  |
| 9.94.010(2) | PRISON RIOT-PARTICIPATE/AID/ABET PENALTY |  | x |  |
| 9A.36.031 | ASSAULT-3 |  | x |  |
| 9A.36.031(1)(A) | ASSAULT-3 RESIST PROCESS/APPREHENSION |  | x |  |
| 9A.36.031(1)(B) | ASSAULT-3 TRANSIT EMPLOYEE |  | x |  |
| 9A.36.031(1)(C) | ASSAULT-3 SCHOOL BUS DRIVER/MECHANIC |  | x |  |
| 9A.36.031(1)(D) | ASSAULT-3 BODILY HARM |  | x |  |
| 9A.36.031(1)(E) | ASSAULT-3 FIREFIGHTER |  | x |  |
| 9A.36.031(1)(F) | ASSAULT-3 SUBSTANTIAL PAIN |  | x |  |
| 9A.36.031(1)(G) | ASSAULT-3 LAW ENFORCEMENT OFFICER |  | x |  |
| 9A.36.031(1)(H) | ASSAULT-3 OFFICER PROJECTILE STUN GUN |  | x |  |
| 9A.36.031(1)(I) | ASSAULT-3 HEALTH CARE WORKER |  | x |  |
| 9A.36.041 | ASSAULT-4 |  | x |  |
| 9A.36.041(1) | ASSAULT-4 |  | x |  |
| 9A.36.041(2) | ASSAULT-4 |  | x |  |
| 9A.36.041(3) | ASSAULT-4 PRIOR DV |  | x |  |
| 9A.36.041(4) | ASSAULT-4 DV DEF FAMILY/HOUSEHOLD |  | x |  |
| 9A.36.045 | DRIVE-BY SHOOTING |  | x | x |
| 9A.36.070 | COERCION |  | x |  |
| 9A.36.080 | MALICIOUS HARASSMENT |  | x |  |
| 9A.36.080(1)(A) | COMMISSION OF HATE CRIME INJURY TO PERSON |  | x |  |
| 9A.36.080(1)(C) | MALICIOUS HARASSMENT PERSON/GROUP |  | x |  |
| 9A.36.090 | THREATS TO GOVERNOR OR FAMILY |  | x |  |
| 9A.36.100 | CUSTODIAL ASSAULT |  | x |  |
| 9A.36.100(1)(B) | CUSTODIAL ASSAULT |  | x |  |
| 9A.36.140 | ASSAULT OF CHILD-3 |  | x |  |
| 9A.36.150 | INTERFERING-DOMESTIC VIOLENCE REPORTING |  | x |  |
| 9A.40.020 | KIDNAPPING-1 |  | x |  |
| 9A.40.020(1)(B) | KIDNAPPING-1 COMMISSION FELONY/FLIGHT |  | x |  |
| 9A.40.020(1)(C) | KIDNAPPING-1 BODILY INJURY INTENT |  | x |  |
| 9A.40.030(1) | KIDNAPPING-2 |  | x |  |
| 9A.40.040(1) | UNLAWFUL IMPRISONMENT |  | x |  |
| 9A.40.060 | CUSTODIAL INTERFERENCE-1 |  | x |  |
| 9A.40.070(2) | CUSTODIAL INTERFERENCE-2 PARENT |  | x |  |
| 9A.40.090 | LURING |  | x |  |
| 9A.42.100 | ENDANGERMENT WITH CONTROLLED SUBSTANCE |  | x |  |
| 9A.44.060 | RAPE-3 |  | x |  |
| 9A.44.060(1)(A) | RAPE-3 NO CONSENT |  | x |  |
| 9A.44.079 | RAPE OF A CHILD-3 |  | x |  |
| 9A.44.083(1) | CHILD MOLESTATION-1 |  | x |  |
| 9A.44.086 | CHILD MOLESTATION-2 |  | x |  |
| 9A.44.089 | CHILD MOLESTATION-3 |  | x |  |
| 9A.44.093 | SEXUAL MISCONDUCT W/MINOR-1 |  | x |  |
| 9A.44.096 | SEXUAL MISCONDUCT W/MINOR-2 |  | x |  |
| 9A.44.100(1) | INDECENT LIBERTIES NOT FORCIBLE COMPULSION |  | x |  |
| 9A.44.100(1)(A) | INDECENT LIBERTIES-FORCE |  | x |  |
| 9A.44.100(1)(B) | INDECENT LIBERTIES-INCAPABLE CONSENT |  | x |  |
| 9A.44.100(1)(D) | INDECENT LIBERTIES-HEALTH CARE PROVIDER |  | x |  |
| 9A.44.160(1) | CUSTODIAL SEXUAL MISCONDUCT-1 |  | x |  |
| 9A.44.170 | CUSTODIAL SEXUAL MISCONDUCT-2 |  | x |  |
| 9A.46.020.2BIII | HARASSMENT-CRIMINAL JUSTICE PARTICIPANT |  | x |  |
| 9A.46.020(1) | HARASSMENT GM |  | x |  |
| 9A.46.020(1)(A) | HARASSMENT KNOWNGLY THREATEN |  | x |  |
| 9A.46.020(1)AI | HARASSMENT-THREATEN BODILY INJURY GM |  | x |  |
| 9A.46.020(2)(A) | HARASSMENT |  | x |  |
| 9A.46.020(2)(B) | HARASSMENT-PREVIOUS CONVICTION DEATH THREAT FELON |  | x |  |
| 9A.46.020(2)BI | HARASSMENT PREVIOUS CONVICTION |  | x |  |
| 9A.46.020(2)BII | HARASSMENT THREATEN TO KILL |  | x |  |
| 9A.46.040 | HARASSMENT-COURT ORDERED REQUIRE GM |  | x |  |
| 9A.46.080 | ORDER RESTRICT CONTACT VIOLATION |  | x |  |
| 9A.46.110(1) | STALKING-GROSS MISD |  | x |  |
| 9A.46.110(5)(A) | STALKING PENALTY GROSS MISD |  | x |  |
| 9A.46.110(5)(B) | STALKING-PREVIOUS/ ORDER/ WEAPON/ VICTIM PENALTY |  | x |  |
| 9A.48.020 | ARSON-1 |  | x |  |
| 9A.48.020(1)(A) | ARSON-1 DANGER TO LIFE |  | x |  |
| 9A.48.020(1)(B) | ARSON-1 DAMAGE DWELLING |  | x |  |
| 9A.48.020(1)(C) | ARSON-1 HUMAN IN BUILDING |  | x |  |
| 9A.48.030(1) | ARSON-2 |  | x |  |
| 9A.52.020(1)(B) | BURGLARY-1 ASSAULT |  | x |  |
| 9A.56.120 | EXTORTION-1 |  | x |  |
| 9A.64.020(1) | INCEST-1 |  | x |  |
| 9A.64.020(2) | INCEST-2 |  | x |  |
| 9A.72.110 | INTIMIDATING A WITNESS |  | x |  |
| 9A.72.110(1)(D) | INTIMID WITNESS-NOT REPORT CHILD ABUSE |  | x |  |
| 9A.72.110(2) | INTIMIDATING A FORMER WITNESS |  | x |  |
| 9A.72.160 | INTIMIDATING A JUDGE |  | x |  |
| 9A.76.180 | INTIMIDATING PUBLIC SERVANT |  | x |  |
| 7.94.120(2)GM | UNLAWF FIREARM POSSESSION EXT RISK |  |  | x |
| 77.15.460 | USE/POSS OF LOADED FIREARM |  |  | x |
| 9.41.040.2A.A | FIREARM POSSESS UNLAWFUL-2 ATTEMPT |  |  | x |
| 9.41.040.2A.III | FIREARM POSSESSION UNL-2 U18 |  |  | x |
| 9.41.040(1)(A) | FIREARM POSSESSION UNL-1 |  |  | x |
| 9.41.040(1)(B) | FIREARM POSSESSION UNL-1 PENALTY |  |  | x |
| 9.41.040(2)(A) | FIREARM POSSESSION UNL-2 |  |  | x |
| 9.41.040(2)(B) | FIREARM POSSESSION UNL-2 PENALTY |  |  | x |
| 9.41.050.1A | CARRY CONCEALED PISTOL W/OUT PERMIT |  |  | x |
| 9.41.050.2A | PISTOL-LOADED IN VEHICLE W/O PERMIT |  |  | x |
| 9.41.050(1)(A) | CARRY CONCEALED PISTOL-NO LICENSE |  |  | x |
| 9.41.050(2)(A) | PISTOL-LOADED IN VEHICLE |  |  | x |
| 9.41.050(3)(A) | PISTOL-UNLOADED IN VEHICLE |  |  | x |
| 9.41.080 | DELIVER PISTOL-MINORS & OTHERS |  |  | x |
| 9.41.113 | FIREARM TRANSFER/SALE VIOLATION |  |  | x |
| 9.41.115 | FIREARM TRANSFER/SALE SUBSEQNT VIO |  |  | x |
| 9.41.140 | PISTOL-ALTER IDENTIFYING MARKS |  |  | x |
| 9.41.170 | ALIEN'S LICENSE TO CARRY FIREARMS |  |  | x |
| 9.41.171 | ALIEN UNLAWFUL POSSESS FIREARM |  |  | x |
| 9.41.190(1) | UNLAWFUL FIREARMS |  |  | x |
| 9.41.240 | USE OF FIREARMS BY MINOR |  |  | x |
| 9.41.240(2) | PISTOL POSESS 18-20 YO VIOLATION |  |  | x |
| 9.41.804 | FAIL FILE PROOF SURRENDER FIREARM |  |  | x |
| 9A.56.300(1) | THEFT OF A FIREARM |  |  | x |
| 9A.56.310 | POSSESSION OF A STOLEN FIREARM |  |  | x |
| 9A.76.023(2)(B) | DISARM LAW OFC GUN DISCHRG PEN |  |  | x |

RCW = Revised Code of Washington; UCR = Uniform Crime Reporting

^a^UCR violent crime includes: murder, non-negligent homicide, forcible rape, robbery, and aggravated assault

^b^Non-UCR violent crime includes: intimidation, harassment, threats, violation of protection order, and other crimes that do not fall in the UCR category but reflect the World Health Organization’s definition of violence: “The intentional use of physical force or power, threatened or actual, against oneself, another person, or against a group or community, that either results in or has a high likelihood of resulting in injury, death, psychological harm, maldevelopment or deprivation.”(1)

Supplementary Table 2. Description of Firearm-Related Themes

| **Category^a^** | **Description** | **Example^b^** |
| --- | --- | --- |
| Explicit verbal threat | Evidence that defendant made verbal firearm-related threats | Defendant stated 'I am going to shoot you all' when confronting ex-spouse at home. |
| Explicit written threat | Evidence that defendant made written firearm-related threats | The defendant sent a text to ex-dating partner stating that they 'have a shot gun with their names [the ex-dating partner and their new partner] on it.' |
| Explicit verbal or written threat | Evidence that defendant made verbal or written firearm-related threats, but it is unclear whether threats were verbal or written | The defendant threatened to 'put a gun to their [the victim's] head and pull the trigger.' |
| Threatened-unknown verbal/written/action | Evidence that defendant made firearm-related threats, but it is unclear whether the threat was verbal, written, or an action | Defendant implied a threat to shoot a neighbor. |
| Shooting/discharge^c^ | Evidence that defendant shot or discharged a firearm in non-hunting incidents |  |
| - Intentionally shot and hit person | Evidence that defendant intentionally shot and hit a person with a firearm | Defendant shot at and hit a person who was bicycling away after the two had an argument. Victim was hit in the forearm. |
| - Intentionally shot but did not hit person | Evidence that defendant intentionally shot at a person with a firearm, but no evidence that the person was hit | The defendant retrieved the firearm from vehicle and began shooting at the victim. |
| - Unintentionally shot and hit person | Evidence that defendant shot and hit a person with a firearm, but the shooting/discharge appears unintentional | Defendant was intoxicated and started arguing with victim. During argument, victim went to grab gun from defendant when the gun went off and fired a bullet through victim's hand. |
| - Intentionally shot non-person but hit person | Evidence that defendant intentionally shot something other than a person (e.g., structure, air) with a firearm, but unintentionally hit a person | Defendant fired pistol into the wall of bedroom for unknown reason. The shot penetrated the wall and neighbor's house, hit neighbor in the leg. |
| - Intentionally shot at non-person, did not hit person | Evidence that defendant intentionally shot something other than a person (e.g., structure, air) with a firearm, no evidence that person was hit | Defendant was in a dispute with neighbors. Defendant fired one 'warning shot' letting neighbors know to get off the property. |
| - Intentionally shot around person, did not hit person | Evidence that defendant intentionally shot a firearm around/near a person, no evidence that person was hit | Defendant fired a shot in victim’s general direction, saying ‘I'm not fucking around.’ |
| - Intentionally shot animal | Evidence that defendant intentionally shot an animal with a firearm | Defendant shot and killed neighbor's dog. |
| Physical harm | Evidence that defendant inflicted physical harm with a firearm that did not involve shooting or discharging | Defendant used a pistol and 'pistol whipped' the victim in the face, striking the victim several times. |
| Point/touch firearm | Evidence that defendant pointed a firearm at a person or touched a firearm to a person’s body | Defendant pointed a gun at the victim's manager during a robbery. |
| Brandish | Evidence that defendant displayed, showed, or waved a firearm, including in pictures/ videos (other than pointing a firearm at a person or touching a firearm to a person’s body) | Defendant followed victim in vehicle and began shouting obscenities and waving a handgun towards victim. |
| Other firearm threat | Evidence that defendant made firearm-related threats (other than brandishing or pointing/touching), e.g., making firearm hand gestures, touching firearm safe, leaving voicemail with firearm sounds, reaching for law enforcement’s firearm | Defendant threatened to kill the victim while using hand to mimic the use of a gun. |
| Hunting | Evidence the defendant violated hunting laws | Gun used to hunt bobcats. |
| Unlawful possession | Evidence that the defendant unlawfully possessed a firearm (e.g., because prohibited person, under legal age of purchase, possession of stolen firearm) | Defendant carried a loaded pistol in a vehicle and did not have a concealed pistol license. |
| Possession | Evidence that defendant possessed a firearm during the alleged criminal incident | Defendant was armed with a handgun and was threatening to shoot/kill the victim. |
| Stole firearm | Evidence that defendant stole a firearm | Defendant stole multiple firearms from a residence. |

^a^Categories are not mutually exclusive per case

^b^De-identified summaries of probable cause narratives

^c^Sub-categories of shooting/discharge are mutually exclusive

Supplementary Table 3. Sensitivity and Negative Predictive Value of Firearm-Related Cases With >=1 Charge for Murder, Non-Negligent Homicide, or Assault in the 1^st^ or 2^nd^ Degrees

|  |  | Manual Review | | |  |
| --- | --- | --- | --- | --- | --- |
|  |  | Firearm+ | Firearm- | Total |  |
| Criminal Codes | Firearm+ | 18 | 0 | 18 |  |
|  | Firearm- | 75 | 759 | 834 | NPV=91.0% (95% CI=88.9-92.9%) |
|  | Total | 93 | 759 | 852 |  |
|  |  | Se=19.4% (95% CI=11.9-28.9%) |  |  |  |

Se = Sensitivity; NPV = Negative Predictive Value

Supplementary Table 4. Sensitivity and Negative Predictive Value of Firearm-Related Cases With >=1 Charge for Robbery in the 1^st^ or 2^nd^ Degrees

|  |  | Manual Review | | |  |
| --- | --- | --- | --- | --- | --- |
|  |  | Firearm+ | Firearm- | Total |  |
| Criminal Codes | Firearm+ | 1 | 0 | 1 |  |
|  | Firearm- | 14 | 95 | 109 | NPV=87.2% (95% CI=79.4-92.8%) |
|  | Total | 15 | 95 | 110 |  |
|  |  | Se=7.1% (95% CI=0.2-33.9%) |  |  |  |

Se = Sensitivity; NPV = Negative Predictive Value

Supplementary Table 5. Sensitivity and Negative Predictive Value of Firearm-Related Cases With >=1 Charge for Rape in the 1^st^ or 2^nd^ Degrees

|  |  | Manual Review | | |  |
| --- | --- | --- | --- | --- | --- |
|  |  | Firearm+ | Firearm- | Total |  |
| Criminal Codes | Firearm+ | 0 | 0 | 0 |  |
|  | Firearm- | 1 | 35 | 36 | NPV=97.2% (95% CI=85.5-99.9%) |
|  | Total | 1 | 35 | 36 |  |
|  |  | Se=0% (95% CI=0-97.5%) |  |  |  |

Se = Sensitivity; NPV = Negative Predictive Value

References

1. Krug EG, Mercy JA, Dahlberg LL, Zwi AB. The world report on violence and health. Lancet Lond Engl. 2002 Oct 5;360(9339):1083–8.
